# Estimating System State through Similarity Analysis of Signal Patterns

**DOI:** 10.3390/s20236839

**Published:** 2020-11-30

**Authors:** Kichang Namgung, Hyunsik Yoon, Sujeong Baek, Duck Young Kim

**Affiliations:** 1 Department of Mechanical Engineering, Ulsan National Institute of Science and Technology, Ulsan 44919, Korea; kichang@unist.ac.kr; 2 Department of Industrial and Management Engineering, Pohang University of Science and Technology, Pohang 37673, Korea; hyunsikyoon@postech.ac.kr; 3 Department of Industrial Management Engineering, Hanbat National University, Daejeon 34158, Korea; sbaek@edu.hanbat.ac.kr

**Keywords:** fault detection, state prediction, pattern extraction, similarity analysis

## Abstract

State prediction is not straightforward, particularly for complex systems that cannot provide sufficient amounts of training data. In particular, it is usually difficult to analyze some signal patterns for state prediction if they were observed in both normal and fault-states with a similar frequency or if they were rarely observed in any system state. In order to estimate the system status with imbalanced state data characterized insufficient fault occurrences, this paper proposes a state prediction method that employs discrete state vectors (DSVs) for pattern extraction and then applies a naïve Bayes classifier and Brier scores to interpolate untrained pattern information by using the trained ones probabilistically. Each Brier score is transformed into a more intuitive one, termed state prediction power (SPP). The SPP values represent the reliability of the system state prediction. A state prediction power map, which visualizes the DSVs and corresponding SPP values, is provided a more intuitive way of state prediction analysis. A case study using a car engine fault simulator was conducted to generate artificial engine knocking. The proposed method was evaluated using holdout cross-validation, defining specificity and sensitivity as indicators to represent state prediction success rates for no-fault and fault states, respectively. The results show that specificity and sensitivity are very high (equal to 1) for high limit values of SPP, but drop off dramatically for lower limit values.

## 1. Introduction

In recent years, products and industrial systems have increased in complexity due to the implementation of various technologies for meeting diverse demands. The possibility of unexpected faults has also increased, potentially resulting in loss of brand value. Hence, a number of maintenance strategies have been introduced for assuring system reliability [1]. Among these strategies, condition-based maintenance (CBM) has been widely applied in industry owing to its low-cost advantage over conventional maintenance strategies [2]. CBM reduces costs by conducting maintenance tasks only when abnormal system behavior is detected. Detection of abnormal behaviors is carried out using information extracted through condition monitoring. In general, condition monitoring involves various methods for extracting the relationship between sensor data and the state of the physical system [3]. Model-based fault detection approaches aim to describe the actual status of a system by mathematical system equations. Furthermore, several graphical formal models such as automata, petri net, and bond graph have been developed to predict faults in electro-mechanical systems [4]. To do this, collected operational data are analyzed as a directed graph where nodes indicate each step or operation of a system and edges represent the directions of system step transitions between two connected nodes. When the given operational data include distinguishable discrete and timed events, graphical model-based approaches provide high fault detection performances. Mathematical equations including Linear Parameter Varying (LPV), quasi-LPV, and Takagi-Sugeno, were usually used to explain the current status of a closed-loop feedback controller [5]. The well-fitted equation should be determined for a target system, and residual signals can then be calculated as fault indicators. However, it is usually not straightforward to develop a precise system model in such a way that it can elicit residual signals as sensitive as possible to faulty behavior of the system, but not sensitive to any system disturbance. Therefore, statistical distance-based methods such as Statistical Process Control (SPC) have been widely applied in practice, and they are representative condition monitoring methods. Specifically, such methods detect and create alerts for system’s abnormalities by measuring statistical distances between the features of trained models and a current data [6]. Principal Component Analysis (PCA) has been popularly used for dimension reduction of high-dimensional datasets. Salo et al. [7] proposed a hybrid dimension reduction technique that combines an information gain approach for discarding irrelevant features and PCA for finding an optimum subset of attributes. Wang et al. [8] presented a fault diagnosis strategy based on PCA and multiclass relevance vector machine for a Cascaded H-Bridge Multilevel Inverter System. They demonstrated the efficiency of fault diagnosis by employing PCA-based dimensional reduction. Zhang et al. [9] combined a model-based approach and statistical detection method, i.e., state observer and PCA, to diagnose fault states in an actuator of multi-propeller aerostats. First, they used a PCA model to detect fault occurrences in real time, and then applied a linearization model to identify small disturbance of a system. Finally, location and severity of a detected fault were calculated using a state observer model.

However, recent advances in sensor technologies have increased the number and variety of sensors installed in manufacturing systems to enable more informative interpretation in condition monitoring. Simultaneously, the difficulty of direct interpretation of such multivariate data using the statistical distance-based method has increased due to high complexity and computational cost [10]. Therefore, a number of condition-monitoring methods have been proposed that condense the dimension of the data while retaining sensitivity to meaningful features that relate strongly to the system’s physical state.

Pattern recognition has been used for extracting comprehensive information from the data for such complex systems, particularly multivariate data [11,12]. Pattern recognition-based methods, which include autoregressive model analysis [13], piecewise aggregate approximation [14], and symbolic aggregate approximation [15], consider the data as a collection of discrete events via piecewise discontinuous functions. Data discretization, which divides a continuous time series into discrete segments and specifies relevant labels to each segment, is a popular technique for such symbolic representations. In addition, symbolic representation of the data provides the practical advantages of high computational efficiency and reduced sensitivity to measurement noise [16,17].

Anomaly detection based on Markov chains has been applied to structural fatigue damage prediction using symbolic patterns extracted from ultrasonic sensor data [18]. Another approach, which generates discrete state vectors (DSVs) to represent the system state by unifying the symbolic patterns of multi-sensor data, has been experimentally validated for condition monitoring of vehicle engines, engines of ships, and laser welding processes [19]. All these methods determine the state of the system based on the distance of patterns of the given data from trained patterns of no-fault state data. Euclidian distance, Mahalanobis distance, Shannon’s entropy, and Kullback-Leibler divergence are popular methods for calculating the distance between patterns [20,21].

Trained patterns cannot perfectly represent all states of the system, however, since real-world data are quite limited. Thus, such methods may not properly determine the state of the system when untrained patterns are extracted. In this case, distance-based pattern classifiers would determine the state by identifying the pattern from a trained-pattern library that is most similar to the untrained pattern. However, distance-based pattern classifiers cannot guarantee correctness of classification when applied to high-dimension multivariate data. Extraction of patterns from multivariate data increases the number of patterns that cannot be trained, since the complexity of the data is increased. For instance, when patterns extracted from different states of the system have the same distance from an unknown pattern, distance-based classifiers cannot clearly determine the system state.

Inspired by this weakness of distance-based classifiers, this paper proposes a new pattern classifier that can robustly classify the system states with multivariate data, even though the given pattern is not trained. To do so, the proposed method applies a probabilistic approach and scoring method to supplement information about the untrained pattern. We call this the “state prediction method” since this paper aims to estimate the state of an electromechanical system using multivariate data. In the remainder of this paper, the detailed procedure of the proposed method is presented with a case study to evaluate its performance.

## 2. State Prediction Method

**Figure 1:**
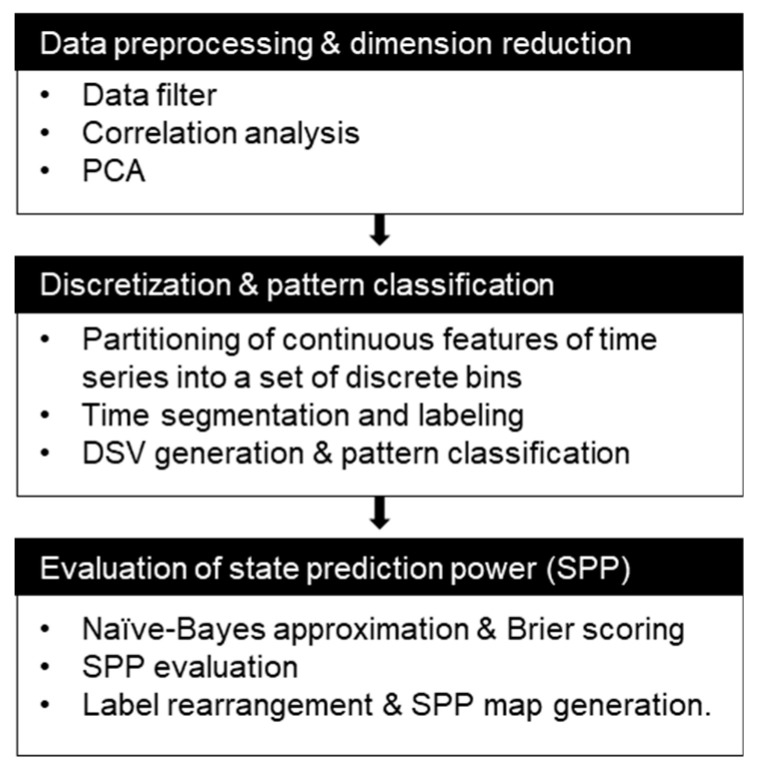
Overall procedure of state prediction.

This section presents the three-step procedure of system state prediction as shown in Figure 1. First, raw sensor signals are conditioned and filtered to reduce measurement noise. PCA-based dimension reduction for multi-sensor signals is conducted. In some cases of data-driven fault diagnostics, a very limited amount of fault occurrences due to the recent high reliable systems have hindered significant pattern identification despite a large amount of collected sensor data, a so-called data imbalance problem. For this case, it is sometimes necessary to utilize every available sensor information for pattern mining. Among these sensors, more informative sensors or principal components can be extracted by dimension reduction techniques.

Second, discretization is made by time segmentation and value range partitioning for the shortlisted sensors or chosen Principal Components (PCs). DSV is then obtained in such a way that it describes the state of a system in a specific time segment. Fault patterns are extracted by analyzing the DSVs. Finally, the state prediction power of each pattern is evaluated based on naïve Bayes approximation and probabilistic scoring rule.

### 2.1. Pattern Definition Using Discretized State Vector

**Figure 2:**
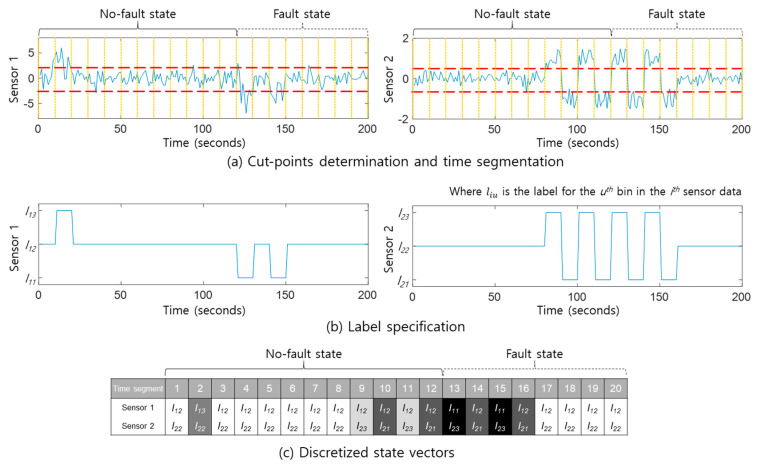
Transformation of multi-sensor signals to a series of discretized state vectors [19].

Before introducing the proposed method in detail, it is necessary to define the characteristics of the pattern used. The concept of DSVs from previous research [19] is applied to pattern extraction from multi-sensor signals owing to its simple representation of multivariate time series data, as shown in Figure 2. Assume that *m* sensor signals are acquired from a target system, from 1 to *n* measurements (i.e., the length of time series of each sensor signal), then it can be written as a matrix X, and its sizes of row and column are *m* and *n*, respectively. Time series segmentation with a fixed length of time window, i.e., *w* measurements, is made for data discretization as illustrated by yellow vertical lines in Figure 2a.

Sensor values are subdivided into a predefined number *k* of bins for data digitization. For example, three bins are specified by two cut-points, red dotted lines in Figure 2a. In this study, the equal-frequency binning method was applied for cut-point determination as follows: first, every sensor data is sorted in ascending order, and the cut-points are then determined so that the number of measurements included in each bin is identical [22]. The appropriate labels are finally allocated to each bin by considering the value of each feature in the sensor signal. For example, $l_{12}$ is the label for the second bin of Sensor 1 in Figure 2b. The average value of *w* measurements in a time segment is used for label specification. Figure 2c shows a series of 20 DSVs for two sensor signals. In other words, a DSV is a vector consisting of all the sensor labels in a time segment.

In this way, the time series for the multivariate data is transformed into a set of DSVs. The DSV is regarded as the pattern for the purposes of the proposed method. It is important to note that DSVs that only occur in the no-fault state are defined as no-fault patterns, while DSVs that only occur in fault states of the system are defined as fault patterns.

### 2.2. Probabilistic Scoring Rule for State Prediction

Using the construction of DSVs outlined above, the proposed state prediction method aims to classify the system state when the DSVs are trained with a small amount of data that is insufficient to account for all the behaviors of the system. The state prediction method is designed to compensate for the lack of prior information by probabilistically calculating a score that represents the relationship between DSVs and given states of the system [23]. Each sensor’s characteristics are assumed to be independent of all the others, since the sensors are physically isolated from each other. Under this assumption, a naïve Bayes classifier is applied that considers the independence of the elements to be classified. Naïve Bayes classifiers have been applied to state prediction for mechanical systems such as centrifugal pumps [24] and three-phase induction motors [25].

The first step in applying a naïve Bayes classifier with DSVs is calculating the prior information of each state from the training data. Consider a system consisting of *n* sensors. Let *N* and *F* be the sets of no-fault and fault-state DSVs, respectively; let $V_{m}={\left[l_{1m_{1}},\;l_{2m_{2}},\ldots ,l_{n,m_{n}}\right]}^{T}$ be the *m*th DSV of the given system, while $l_{i,m_{i}}$ is the determined label for the *m*th time segment in the *i*th sensors data (*i* =1, 2, …, n).

P(N) and P(F) represent the probabilities of a no-fault and a fault pattern, respectively. When the probabilities are not given, they are estimated by calculating the ratio of observations of each state to the total number of observations. For example, if the ratios of the fault and no-fault states of the given data are 70% and 30%, the probabilities are assumed to be 0.7, and 0.3, respectively.

Next, conditional probabilities of fault and no-fault states given observed DSVs are calculated for the naïve Bayes classifier. $P\left(F|V_{m}\right)$ is the conditional probability of a fault state given $V_{m}$, while $P\left(V_{m}|F\right)$ is the conditional probability of $V_{m}$ given a fault state. According to Bayes’ theorem,

(1) $$P\left(F|V_{m}\right)=\frac{P\left(V_{m}|F\right)\times P\left(F\right)}{P\left(V_{m}|F\right)\times P\left(F\right)+P\left(V_{m}|N\right)\times P\left(N\right)}$$

where $P\left(N|V_{m}\right)$ is defined as the remainder of subtracting $P\left(F|V_{m}\right)$ from 1:

(2) $$P\left(N|V_{m}\right)=1-P\left(F|V_{m}\right).$$

$P\left(V_{m}\vee F\right)$ is calculated by multiplying the conditional probabilities of each label in $V_{m}$, given a fault state:

(3) $$P\left(V_{m}\vee F\right)=P\left(l_{1,m_{1}}|F\right)\times P\left(l_{2,m_{2}}|F\right)\times \ldots \times P\left(l_{n,m_{n}}|F\right).$$

**Figure 3:**
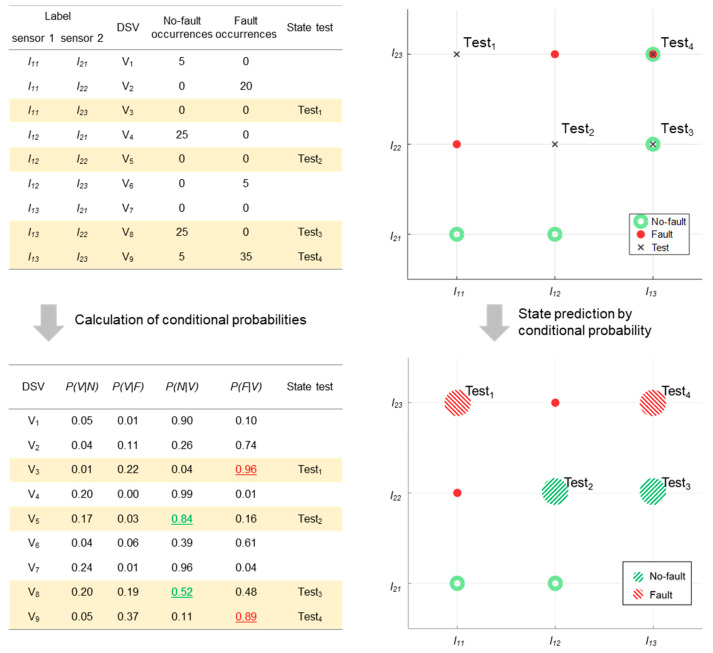
State prediction by conditional probability.

In addition, Laplace smoothing is applied to prevent the conditional probability from becoming zero before calculating the likelihoods of the DSVs. The probabilities are calculated by assuming the occurrence of the unobserved DSVs to have probabilities summing to 1. Figure 3 shows an example of calculating the conditional probabilities based on the observations as well as the state prediction procedure using these probabilities. The dataset consists of two sensors, and each sensor’s data are discretized with three labels. Therefore, nine DSVs in total can be generated and their corresponding conditional probabilities are obtained using Equations (1)–(3).

The states of the four test DSVs, named Test_1_, Test_2_, Test_3_, and Test_4_, are classified using the obtained probabilities. Test_1_ and Test_4_ are classified as fault states, while Test_2_ and Test_3_ are classified as no-fault states. Although Test_3_ is classified as no-fault, there is no significant difference between $P\left(N|V_{8}\right)$, and $P\left(F|V_{8}\right)$. While the observation table shows that $V_{8}$ only appears in the no-fault state, the appearance of each label or discretized component made the probabilities of both states similar.

This is caused by taking into account the independence of each label when calculating the conditional probabilities. Hence, the probabilities are more influenced by state bias of each label in the DSVs than by state bias of the DSVs themselves.

Therefore, our state prediction rule needs to consider the error between the prediction and the actual state. The concept of a probabilistic scoring rule has been applied to distortion-free calculation of this error based on probabilistic assurance. Among the various scoring methods, the Brier score has been adopted in much research in order to emphasize the relationship between the system state and each DSV [26]. The sum of the squared prediction errors for the entire set of predictions is calculated, while the actual state is known [27]. The definition of the Brier score is as follows:

(4) $$BS=\frac{1}{T}\overset{T}{\underset{i=1}{\sum }}{\left(p_{i}-\delta_{i}\right)}^{2}$$

where T represents the total number of prediction events, $p_{i}$ denotes the probability of the ith prediction, and $\delta_{i}$ describes the actual event corresponding to the ith prediction. The value of $\delta_{i}$ is set to 1 for the event predicted by $p_{i}$, and 0 otherwise [28]. The original scoring rule, which is negatively oriented, is given a positive orientation by subtracting the Brier Score from 1 so as to allow more intuitive insight [29].

**Figure 4:**
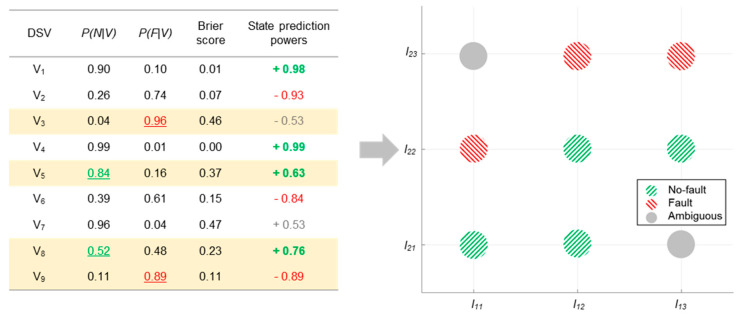
State prediction by state prediction powers.

As shown in Figure 4, the Brier score of a DSV that correctly predicts the state is proportional to the conditional probability of the DSV given that state. On the other hand, the Brier score of a DSV that incorrectly predicts the state is inversely proportional to the conditional probability of the DSV given that state. The Brier scores of DSVs are thus converted into state prediction power (SPP) values, defined for more intuitive decision making. SPP is defined in the following Equations:

(5) $$SPP_{no-fault}=\left(1-BS\right)$$

(6) $$SPP_{fault}=\left(BS-1\right)$$

where SPP values range from −1 to 1. The state decision is accomplished by comparing the SPP of the given DSV with a predefined SPP limit value. The limit values for $SPP_{no-fault}$ and $SPP_{fault}$ are predefined so as to have the same absolute values but opposite signs. For example, if the limit value for $SPP_{fault}$ is defined as −0.6, that of $SPP_{no-fault}$ is defined as 0.6. The state prediction of the given DSV is considered to be reliable only if $SPP_{fault}$ is lower than −0.6 or $SPP_{no-fault}$ is higher than 0.6.

Figure 4 shows the SPP values that are calculated from the Brier scores of the DSVs. Among the DSVs, $V_{8}$ is determined as a fault DSV when the value of SPP is compared with the limit value, though it was determined as an ambiguous DSV based on the naïve Bayes classifier. However, $V_{3}$, and $V_{7}$ are determined as ambiguous DSVs when comparing their SPP values, even though they were determined as fault and no-fault DSVs, respectively, with the naïve Bayes classifier.

**Figure 5:**
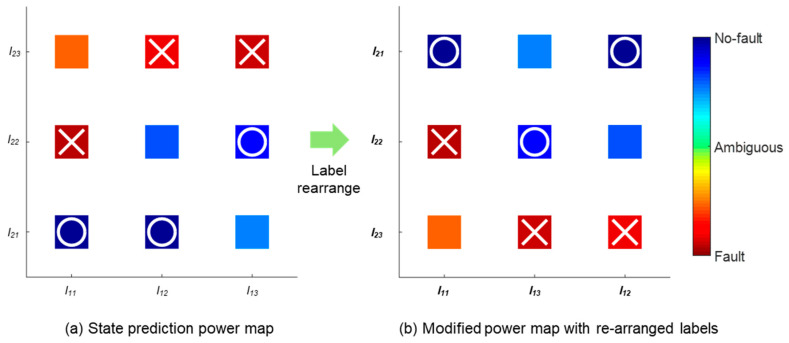
State prediction power map and label rearrange for fault region visualization.

After obtaining the SPPs of all the DSVs, a visualization of the state prediction, termed the state prediction power map, is suggested as Figure 5a. The map is rearranged according to the SPP values for the DSVs as Figure 5b, providing more intuitive information. The no-fault and fault state DSVs that are determined as reliable are marked with O and X, respectively.

## 3. Similarity Analysis by State Prediction Power

### 3.1. Engine Fault Simulator

**Figure 6:**
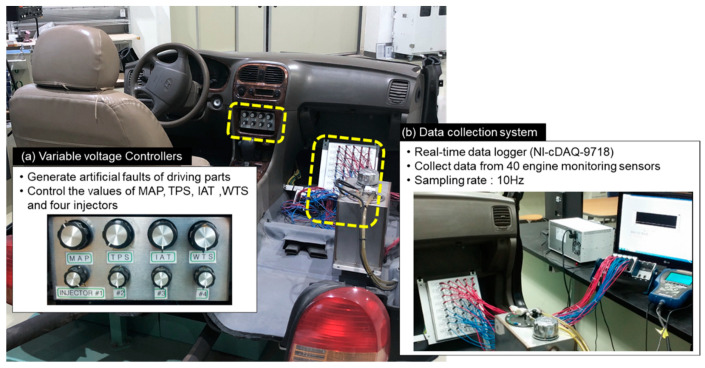
Car engine fault simulator and data collection system: (**a**) variable voltage controllers generate artificial engine faults by changing the gauges of engine components, such as manifold air pressure (MAP), throttle position sensor (TPS), intake air temperature (IAT), water temperature sensor (WTS), and four injectors; (**b**) the data collection system consists of 40 sensors installed at engine components as well as a data acquisition module.

The proposed state prediction method was applied to data collected by a car engine fault simulator. The simulator replicates the occurrence of faults in a car engine by changing sensor values that strongly affect the operating status of the engine. As shown in Figure 6a, no-fault and fault system states are artificially generated by changing the following sensor values: manifold air pressure (MAP), throttle position sensor (TPS), intake air temperature (IAT), water temperature sensor (WTS), and four injectors. Figure 6b shows the data collection system, which consists of 40 sensors, and the data acquisition module (NI-DAQ). Sensor data were collected at a sampling rate of 10 Hz with accuracy of 0.04% gain error in room temperature, timing accuracy of 50 ppm of sample rate, and timing resolution of 12.5 ns. A single analog-to-digital converter of NI-DAQ samples multiple sensor signals through scanning, buffering, and conditioning processes to minimize measurement noise such as missing signal and abnormal peaks.

The artificial fault generation scenario is to replicate the phenomenon of engine knocking, which is caused by a shortage of fuel in the mix. As is well known, the MAP sensor is installed in the automobile electronic control system, which determines the fuel supply to the engine. Engine knocking is generated when the value of MAP decreases, potentially causing the engine to make a loud knocking or “pinging” noise.

In each trial of the experiment, data collection begins immediately after the engine is turned on. After 240 s of engine operation, the MAP value is decreased for 30 s via the control dial. The MAP value is then increased to the no-fault state for 20 s, and finally the engine is turned off. Time information for each stage is recorded for every trial. In total, 430 experimental trials were performed.

### 3.2. Similarity Analysis

**Figure 7:**
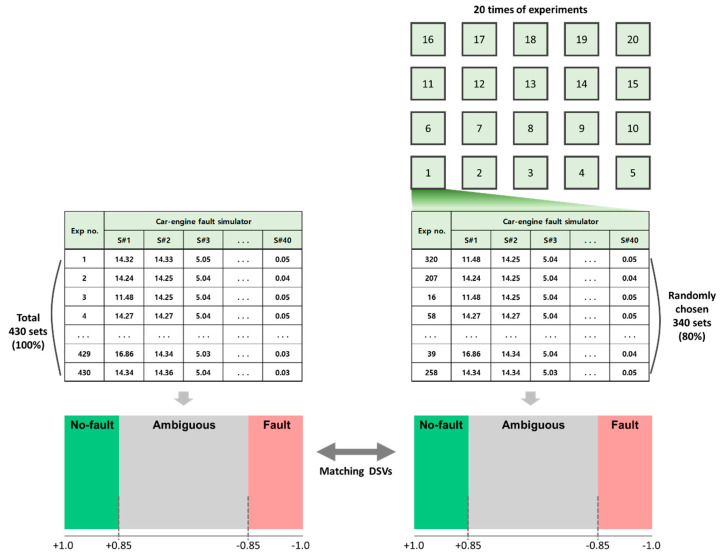
Experiment procedure for 20 repeated experimental trials.

A similarity analysis is performed to demonstrate the effectiveness of the proposed method. The analysis is conducted as follows. First, 6 sensors are selected (out of a total of 40) that have been proven to represent the state of the simulator well. A data set for each sensor is then transformed into a set of DSVs. Repeated holdout cross-validation is applied for estimating the performance of the proposed method, since training data are not given. This study applied holdout rather than k-fold cross-validation since the amount of data is large [30]. Twenty percent of the DSVs are randomly selected as training data, and SPPs are calculated for the data as shown in Figure 7. The remaining DSVs are then classified using the calculated SPP values. The state prediction is performed through 20 repeats of the cross-validation, with the training and test DSVs randomly selected for each trial.

The SPP limit value is set as 0.85 (i.e., the upper and lower limits are set as 0.85 and −0.85). If the SPP for the given DSV is larger than 0.85, the DSV is determined as a reliable state predictor for the no-fault state, and as a reliable state predictor for the fault state when the SPP is lower than −0.85. The responses of the given DSVs can be depicted as shown in Figure 7. “True negative” and “true positive” are the responses when the states of the given DSVs are correctly predicted as no-fault and fault, respectively. A prediction error that judges a no-fault DSV as being in a fault state is called a false alarm (Type I error). A prediction error that assesses a fault-state DSV as being in a no-fault state is called a miss (Type II error). The trial results are summarized in the next section.

## 4. Experimental Results and Discussion

### 4.1. Fault Detection with the SPP

The result of the experimental trials is shown in Table 1. To identify the performance of SPP in fault detection problem, sensitivity and specificity were defined as performance indicators. Sensitivity is defined as the ratio of true positives to the sum of true positives and misses. Specificity, conversely, is defined as the ratio of true negatives to the sum of true negatives and false alarms. In other words, sensitivity and specificity measure the success rate of state prediction for fault and no-fault states, respectively. Therefore, higher value of two indicators indicates higher performance in fault detection.

**Figure 8:**
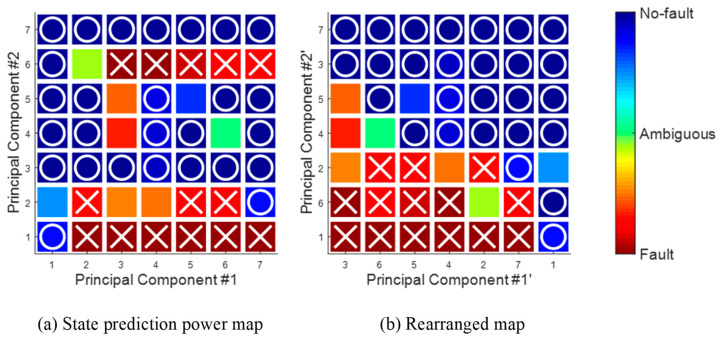
State prediction power map for engine fault simulation data.

As shown in Table 1, both mean values of sensitivity and specificity were the highest, equal to 1 when the limit value is set as 0.85. ‘Min’ in Table 1 indicates the lowest performance within 20 experimental trials, and they mean that a 2.4% miss rate and a 1.5% false alarm rate occurred in a certain validation dataset. A visual representation is provided in the form of a gradual similarity map, which rearranges DSVs according to SPP value for describing the generated state prediction result in more detail. For an intuitive understanding of the concept, Figure 8 shows the two-dimensional state prediction power map generated using DSVs from two sensor signals. SPP values of are placed on a single scale from −1 to +1, represented as a color map. Figure 8a shows 49 DSVs generated using 7 labels. The DSVs are then rearranged according to the values of SPP of each DSVs. This similarity map makes it easier to understand the trend of each state by comparing SPP values of the individual DSVs.

### 4.2. Discussion

**Figure 9:**
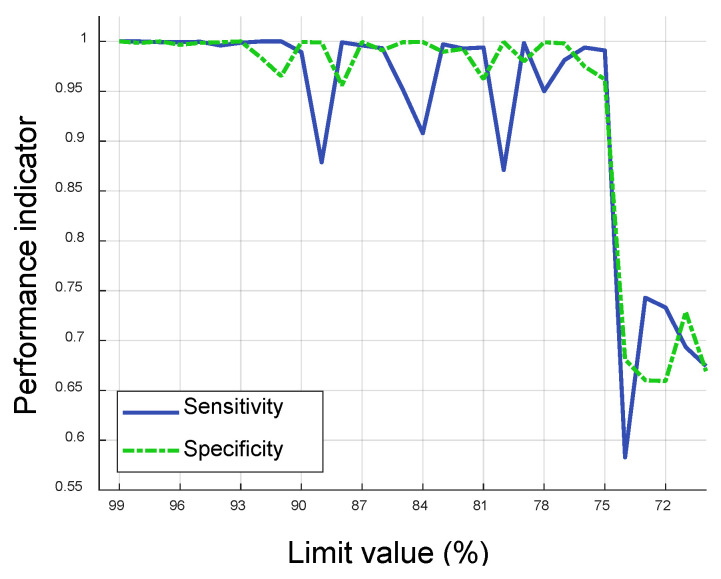
Trends of sensitivity and specificity depending on limit value in fault detection.

In order to analyze the effects of limit values in calculating SPP value, the trends of sensitivity and specificity in fault detection were investigated with respect to the limit value varying from 70% to 99%, and the corresponding fault detection performance was computed as illustrated in Figure 9. Both performance indicators showed a very high level of correct state predictions, specifically near 0.99, when the limit value was set to be above 0.93. Sensitivity varied between 0.88 and 1.0 when the limit was set between 0.75 and 0.93 and specificity showed less variation than sensitivity for the same range of limit values. However, when the limit value was set to be 0.74, both sensitivity and specificity showed dramatic drops, to 0.58 and 0.68, respectively. Both indicators showed values consistently lower than 0.75 when the limit value was lower than 0.74.

It is important to note that two significant principal components (PCs) were extracted through PCA with 40 original sensor signals, and the two PCs were considered as input signals for fault pattern extraction. In general, the generated DSVs from the chosen two PCs are more informative to classify fault and no-fault states compared to the obtained DSVs using raw sensor signals directly. Therefore, from the critical threshold in limit value (i.e., about 75% in Figure 9), ambiguous DSVs which had frequently occurred in both no-fault and fault state began to be observed in fault and no-fault patterns and they made fault detection performance significantly lower. If the original 40 sensor signals are considered, some of which may contain irrelevant features to the systems status change, the gradual drops in sensitivity and specificity can be expected since ambiguous DSVs with different occurrence ratios in fault and no-fault states can be found more uniformly.

In short, this research presented a state prediction method for complex systems that cannot provide large amounts of training time-series data. In particular, the method is useful for the dataset where system faults have rarely occurred or the DSVs of fault patterns were significantly redundant with no-fault ones. Conventional state prediction methods, which use distance-based pattern classifiers, make ambiguous predictions when distances between newly given unknown and trained patterns for system states are the same. Therefore, the state prediction method proposed to interpolate untrained pattern information by using the trained patterns probabilistically. The SPP values represent the reliability of the state prediction, and are obtained by finding the relationship between the conditional probabilities of DSVs given either state and the actual state of the system. As a result, as illustrated in Figure 8a, it is possible to estimate state prediction power of an unfound DSV (for example, $\;\left[\begin{array}{cc} l_{13} & l_{i4} \end{array}\right]$) with regard to conditional probability and an amount of its reliability. It is observed that the estimated prediction power of not-found DSVs was also beneficial to the fault detection problem. In addition, due to rearranging of the labels according to the sum of the SPP value of DSVs on each sensor axis (illustrated in Figure 8b), it is also possible to use not only SPP value but also distance information for identifying the prediction power of signal patterns.

## 5. Conclusions

This research presented the state prediction method for complex systems with imbalanced state data. The proposed method applies naïve Bayes classifier and Brier score to interpolate ambiguous prediction values for unknown or rarely occurred patterns. The computed SPP values for each extracted DSV can be interpreted as the significance of the state prediction. The case study of engine fault simulation showed that both sensitivity and specificity are equal to one with the SPP limit value of 0.85, such that engine knocking was accurately predicted.

In the present study, fault and no-fault binary system states were considered. This was mainly because the system status was marked as fault or normal in the raw sensor data. However, naïve Bayes classifier, by its nature, is not limited to number of states. Therefore, multiple states of system status, for example, type of fault including the severity of system’s failure should be considered for future research. Furthermore, by considering the nature of naïve Bayes approach, the value of SPP is not an exact probability to predict a system’s fault when a DSV is observed in the monitored sensor signals, and hence, probabilistic interpolation should be incorporated with pattern similarity analysis.

## Figures and Tables

**Table 1:** Fault detection results for 20 experimental trials.

Performance Indicator	Decision	
	Sensitivity	Specificity
Mean	1	1
St. Dev.	0.01	0.01
Min	0.98	0.99
